# Sociomaterial perspective as applied in interprofessional education and collaborative practice: a scoping review

**DOI:** 10.1007/s10459-023-10278-z

**Published:** 2023-08-30

**Authors:** Michael Sy, Kathryn Lizbeth Siongco, Roi Charles Pineda, Rainier Canalita, Andreas Xyrichis

**Affiliations:** 1 Institute of Occupational Therapy, Zurich University of Applied Sciences, 8400 Winterthur, Switzerland; 2 College of Nursing, University of the Philippines Manila, 1000 Ermita, Manila, Philippines; 3 Department of Rehabilitation Sciences, Katholieke Universiteit Leuven, 3001 Leuven, Belgium; 4 National Teacher Training Center for the Health Professions, University of the Philippines Manila, 1000 Ermita, Manila, Philippines; 5 School of Physical Therapy, Far Eastern University Nicanor Dr. Nicanor Reyes Medical Foundation, Quezon City, Philippines; 6 Florence Nightingale Faculty of Nursing, Midwifery & Palliative Care, King’s College London, 57 Waterloo Road, 57 Waterloo Road, SE1 8WA UK

**Keywords:** Interprofessional, Sociomateriality, Teaching and learning, Healthcare practice, Review

## Abstract

Learning and working together towards better health outcomes today have become more complex requiring an investigation on how interprofessional education (IPE) and interprofessional collaboration (IPC) practices could be sustained and further developed. Through a sociomaterial perspective, we can better understand IPE and IPC practices by foregrounding the material aspect of learning and working together and examining its relationship with humans and their interactions. This article aimed to examine existing literature that discusses the application of sociomaterial perspectives in IPE and IPC. A scoping review was conducted following Arksey and O’Malley’s framework to explore the extent within the current body of knowledge that discuss how sociomaterial perspective is applied in IPE and IPC practices. A systematic database search was performed in September 2021 to retrieve literature published from 2007 onwards, with forty-three papers meeting the inclusion criteria. These papers included research articles, book chapters, conference papers and commentaries, with the majority originating from Europe. The thematic analysis revealed the following themes: (1) power as a sociomaterial entity shaping IPE and IPC; (2) inclusion of non-health professionals in reimagining IPE and IPC practices, and (3) the critical understanding of sociomateriality. The findings suggest that a sociomaterial perspective can allow for the reimagination of the contemporary and future practices of interprofessionalism.

**Supplementary Information:**

The online version contains supplementary material available at 10.1007/s10459-023-10278-z.

## Introduction

Interprofessional education (IPE) is an internationally endorsed educational reform used in the health and social care professions to promote learning with, from, and about each other to improve collaboration and delivery of care (World Health Organisation, 2010). Learning and assessment in IPE is explicitly recognised in terms of knowledge, skills, attitudes (KSA) or behavioural competencies translated into practice by individuals (Canadian Interprofessional Health Collaborative, 2010). This then developed a platform for interprofessional collaboration (IPC) to occur in the workplace and clinical practice where different health and social care professionals regularly come together and work in teams to negotiate and agree on how to solve complex care problems or provide services, thereby improving health outcomes (Barr et al., 2005; Reeves et al., 2010). With the increasing use of technology in education and health care, IPE and IPC practices have unearthed more complex realities that necessitate further exploration in terms of both theory and application.

The concept *sociomateriality* proposes that organisations (social) and technology (material) are not separate entities but are “inextricably fused” or “constitutively entangled” (Orlikowski & Scott, 2008). Hence, a *sociomaterial perspective* aims to place due attention to the non-human elements and its complex interactions with human elements (Fenwick, 2014a; 2014b). In health professions education and health care practice, there has always been a focus on human-centric views on learning and practice, leaving behind the value of the materials including objects, tools, and technologies that are necessary to make teaching and practice possible. For instance, in the classroom, educators would typically emphasize the importance of the students and their learning, whereas in clinical practice, practitioners would often put focus on the patients, the care given to them, and their overall health outcomes. In these situations, the value of educational materials such as books, technological devices, and specialized medical equipment such as stethoscopes are eclipsed. By foregrounding the material aspects within learning and working together towards better health outcomes for our patients, we are espousing an alternative perspective underpinned by sociomateriality.

IPE and IPC are global reforms promoted to improve delivery of care and health outcomes (World Health Organisation, 2010). However, establishing and enhancing competencies in IPE and IPC continue to be challenging. One of the challenges include educators and practitioners largely focusing on training the individualistic aspect of interprofessional competencies (and therefore, learning) with the belief that competencies are ‘products’ that can be simply acquired (McMurtry et al., 2016). This, in turn, undermines attempts at understanding the complex interplay between materiality and the human-centred elements of learning and practice.

Sociomateriality contributes to further exploring the process of developing knowledge and competencies in IPE and IPC, which are pertained to as “products”, acquired through cognitive and acquisition-oriented learning (McMurtry et al., 2016). Dominant learning theories and “folk assumptions” towards learning, such as behaviourism and cognitivism, may overshadow the value of clarifying IPE and IPC using a sociomaterial lens if these hegemonic learning theories and instructional models remain unexamined and unquestioned (Ertmer & Newby, 1993; McMurtry et al., 2016). Hence, a gap remains in establishing the usability and transferability of IPE and IPC into practice as explained from a sociomaterial perspective.

A comprehensive and updated review about the application of sociomaterial perspective in the context of IPE and IPC can be valuable in informing the adaptation processes within health professions education and health care practice. Thus, the purpose of this article is to examine the literature dealing with the application of sociomaterial perspectives to IPE and IPC. This objective was guided by the research question: “What is known from the existing literature about how sociomaterial perspectives are applied in IPE and IPC?” Such a broad research question allowed for the inclusion of relevant records pertaining to sociomaterial perspectives as applied in IPE and IPC and systematically assess as well as identify the existing body of knowledge on the topic and its gaps (Arksey & O’Malley, 2005; Levac et al., 2010).

## Background

To contextualise this scoping review, the following concepts are discussed: sociomaterial perspectives, interprofessional education, interprofessional learning, interprofessional collaboration, and the intersection of interprofessionalism and sociomateriality.

### Sociomaterial perspectives

The sociomaterial perspective can be traced back to the early works of its main proponent, Wanda Orlikowski (2007). Fenwick et al. (2011) introduced four theories considered as part of the sociomaterial perspective. At that time, these four were argued as the most prominent collective and sociomaterial learning discourses used in IPE and IPC in the field of health care: Cilliers’ (1998) Complexity Science, Engeström’s (2001) Cultural Historical Activity Theory (CHAT), Latour’s (2005) Actor-Network Theory (ANT), and spatiality theories (Fenwick et al., 2011). McMurtry (2013) supported the inclusion of these theories under the umbrella of sociomateriality by reintroducing the same three theories in his 2013 paper, albeit replacing spatiality theories with Lave and Wenger's Communities of Practice (CoP) theory. For the purpose of this article, we use the term ‘sociomaterial(ity) perspectives’ to refer to the theories that are characterised by sociomaterial concepts.

Within the context of higher education, specifically in the teaching and learning of health professionals, learning grounded on a sociomaterial perspective is seen as the enmeshment between the social and material entities, producing the practice of educating, learning, and teaching health professionals. In this situation, *materiality* could refer to non-human elements such as computers, chalk board, marker pens, notes as well as intangible matter like data and algorithms (Leonardi, 2012). On the other hand, *sociality* could refer to the relations between humans including the interaction between teacher and students via videoconferencing or the communication between students via a chat group (MacLeod & Ajjawi, 2020).

Framing the learning and practice of health professionals from a sociomaterial perspective entail the following (Fenwick et al., 2012):

1. Seeing the whole system where human and non-human entities are constitutively entangled, not separate;
2. Shifting the focus from learning and doing as phenomena not only produced by humans but through the equal contribution of both human and non-human elements within the system of knowledge (re)production; and
3. Understanding that practising a profession is the phenomenon that results from sociomaterial connections; therefore, by looking at the actual dynamics between these social and material entities, it can help in examining how systems can be stabilised and destabilised to improve learning and health care practice.

Sociomaterial perspectives have influenced various thinkers, practitioners, and educators (Goldszmidt & Faden, 2016), subsequently extending its reach to health science educationalists who have used the perspective to better understand organisational systems, information management, and further technological advancements. Conversely, critics of sociomaterial perspectives point to the lack of definitive concepts and the overt utilisation of obscure vocabularies through the frequent use of jargons (Parmiggiani & Mikalsen, 2013), gatekeeping its widespread application. Other critics also challenge the notion that the social and material are constitutively entangled. To them, entities may be regarded as related, but can also be mutually exclusive. In other words, the existence and agency of one entity do not depend on the simultaneous existence or actions another. (Leonardi, 2013). The examination of such tensions within health professions education, specifically in IPE and IPC practices, remain sparse. Hence, we seek to translate sociomaterial conceptualisations from philosophical meanderings to pragmatic expressions, bridging the theory–practice gap within the context of IPE and IPC.

### Interprofessional education

While the definition of IPE has been constantly evolving, a commonly accepted definition, developed following an international consensus, refers to IPE as occurring “when members (or students) of two or more health and/or social care professions engage in learning with, from and about each other to improve collaboration and the delivery of care.” (Journal of Interprofessional Care [JIC], n.d., p. 1). IPE is an integral part of health professions education, where learning is inherently social in nature, shifting its focus from the individual to a more holistic and shared learning experience (Oates, 2016). Historically, IPE has been taught and assessed using the traditional instructional model of learning where learners and knowledge are distinct, and that the former must acquire the latter for learning to occur. It is also in this context that team-based learning is assessed using parameters that are focussed on individual learner’s acquisition of interprofessional competencies rather than how individuals acquire competencies as a group or team (McMurtry et al., 2016). Underpinned by a sociomaterial perspective, IPE within the larger system of health professions education can lead educators and learners to reimagine that learning and practice constitute the enmeshment of non-human (e.g., learning materials, technology) and human entities (e.g., students, professionals, educators). For instance, curriculum designers may begin to reformulate learning outcomes not solely based on compartmentalised learning domains (i.e., KSA) but rather on the intersections of these learning domains. Likewise, due emphasis can be designated to the material aspects of learning where instructors intentionally incorporate tangible and intangible learning materials to support the teaching–learning process.

### Interprofessional collaboration

The World Health Organisation (WHO, 2010) describes interprofessional collaboration or IPC as the result of successful IPE that produces collaborative practice-ready health care professionals. Within this context, IPC (or collaborative practice) is defined as: “[An occurrence] when multiple health workers from different professional backgrounds provide comprehensive services by working with patients, their families, carers and communities to deliver the highest quality of care across settings (p. 13).” In addition, “practice” includes both clinical and non-clinical health-related work, such as diagnosis, treatment, surveillance, health communications, management, and sanitation engineering (WHO, 2010).

### Interprofessionalism and sociomateriality

Sociomaterial perspectives, regardless of their philosophical, ontological, and epistemological origins, have the following key notions (Orlikowski & Scott, 2008):

1. *Materiality* emphasis on the importance of matter and the material.
2. *Inseparability* material and social entities are intertwined and cannot be discerned separately.
3. *Relationality* humans and materials only exist in relation to each other.
4. *Performativity* only through the relations of human and material elements can agency be enacted through practice.
5. *Practice* heterogenous gatherings of natural, technological, human, and non-human actors that form assemblages of bodily movements, mental activities, objects and their use, states of emotions, know-how, and motivation, among others.

These terminologies provide a baseline understanding on how human and non-human elements assemble in complex ways within interprofessional interactions. Knowing and not knowing these key notions of sociomateriality enable or constrain, respectively, the development of interprofessional competencies (Goldszmidt & Faden, 2016). Therefore, exploring how sociomateriality principles are being applied in IPE and IPC is pivotal not only because it needs to be translated in practical terms, but also because it is essential to improve teaching and learning within the larger networks and systems of health professions education and health care.

Through the lens of sociomateriality, IPC highlights the main premise that social and material forces, culture, nature and technology are enmeshed in everyday health professional practice (Fenwick et al., 2012; Fenwick, 2014a). This enmeshment is deemed necessary for learning and practising clinical competencies to become or assume roles as a health professional (Fenwick, 2014a). A sociomaterial perspective challenges current practice conventions where health outcomes are determined solely by human agency (Fenwick & Dahlgren, 2015). Shifting attention to the non-human elements and positioning them at the foreground of professional practice can provide health care professionals the opportunity to stabilise efficient assemblages and dismantle deficient ones to inform practice towards achieving better health outcomes. A sociomaterial perspective, however, does not encourage creating a dichotomy between human and non-human elements within medical and health practice rather, as Fenwick (2014a) argues, it is through the strengthening of the interplay between these elements that interprofessional collaboration, coordination, networking, knotworking, and teamworking can be successfully attained.

## Methods

This scoping review was guided by the five-stage framework described by Arksey and O’Malley (2005) with further recommendations from Levac et al. (2010). These stages include: (1) identifying the research question; (2) identifying relevant studies; (3) study selection; (4) charting the data; and (5) collating, summarising and reporting the results. Reporting of this review’s findings was informed by the Preferred Reporting Items for Systematic Reviews and Meta-Analyses—Extension for Scoping Reviews (PRISMA-ScR) (Tricco et al., 2018). Stage 1 was described in the introduction while the subsequent stages are briefly described below. The full study protocol is described in Sy and associates (2022).

### Identifying relevant studies

**Fig. 1:**
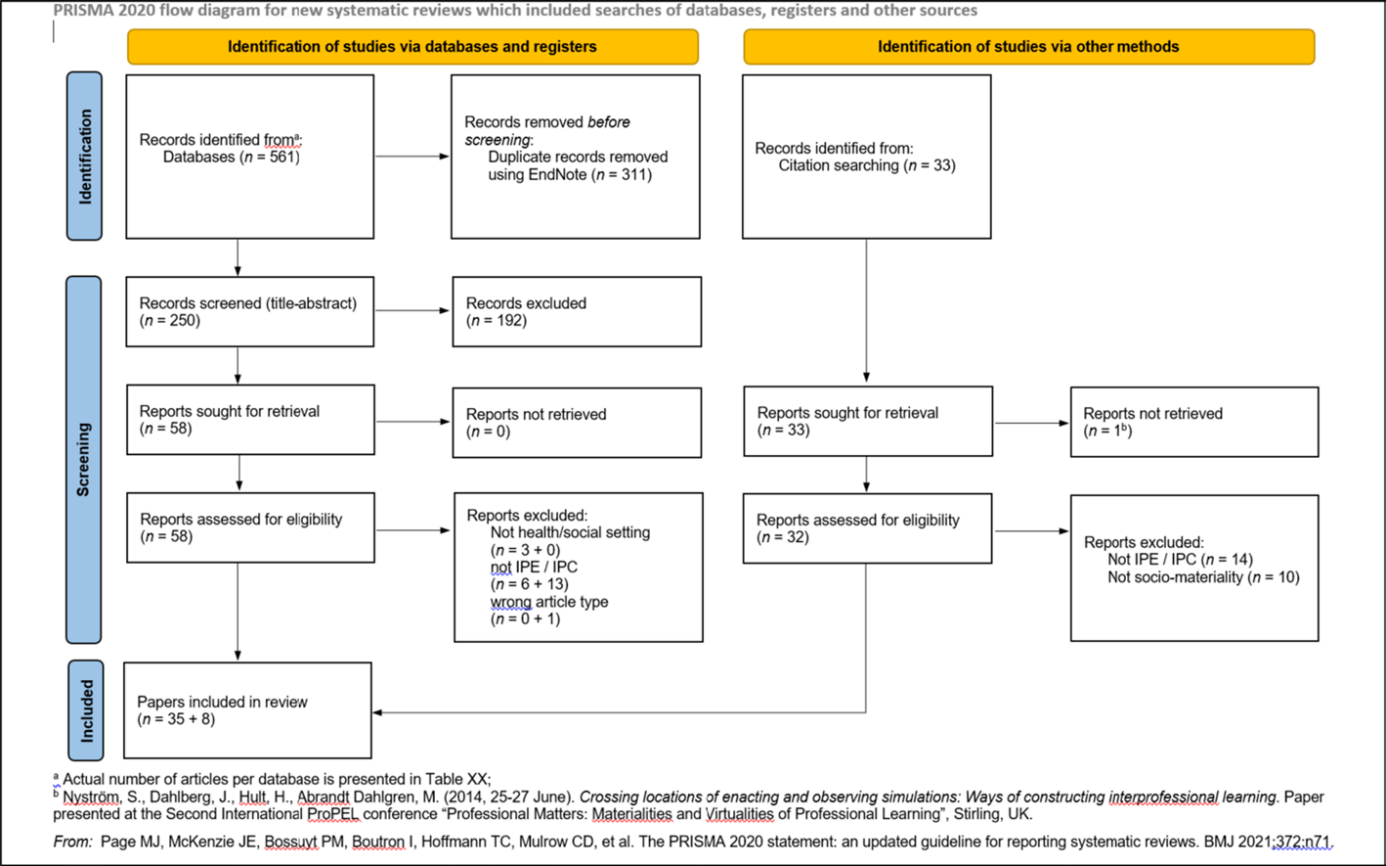
PRISMA diagram

The key terms in the development of a search strategy were ‘interprofessional’ and ‘sociomateriality.’ Although the terms *interdisciplinary*, *multidisciplinary* and *transdisciplinary* are distinct from ‘interprofessional,’ we opted to include these and its other related terms as they are often used interchangeably in the literature (Brewer et al., 2016). Several sociomaterial theories were considered as search terms but were ultimately excluded from the search strategy not only due to distinct variations in foci, approaches, and assumptions across these theoretical traditions (Fenwick, 2014b), but also to manage yield from the database search. Free text search was limited to the title, abstract, and keyword fields and spelling/grammatical variants of key terms were incorporated using the truncation function. When available, database-specific controlled vocabularies were utilised. We ran the search in the following electronic databases: (1) MEDLINE (Ovid); (2) ERIC (Ovid); (3) CINAHL (EBSCO); (4) Embase; (5) Web of Science (Core Collection); (6) Proquest Central; (7) Scopus; and (8) PsycInfo (Ovid). Additionally, the search was limited to literature written in English and those published from 2007 onwards, which marks Orlikowski’s seminal work on sociomateriality. Figure 1 illustrates the search string that we used.

### Selection of sources of evidence

Deduplicated records from the database search were independently screened for eligibility by at least two of three researchers (MPS, KLS, and RFC). Inclusion criteria included: (1) contains the concept of sociomateriality; (2) pertains to the education and/or practice of health and social care, addressing relevant topics on the field of health professions education and research; and (3) includes an element of interprofessionalism following the JIC and WHO definitions. Disagreements between reviewers were resolved through a discussion involving a third reviewer. Finally, we performed a supplementary search by checking the reference list and conducting a cited reference search of all included records using Scopus and Web of Science.

### Charting the data

Records that met the inclusion criteria were summarised using a data charting form that was iteratively developed by the reviewers. The following information was charted: (1) authors; (2) year of publication; (3) country where study was conducted or, when not applicable, the author’s affiliation; (4) definition of sociomateriality used; (5) IPE/IPC setting; (6) professions involved; (7) related theories identified; (8) rationale for adopting socio-materiality; and (9) application of sociomateriality in IPE/IPC. Four authors (MPS, KLS, RFC, and RCSP) performed data charting, with at least two authors charting each included record independently. The researchers convened every ten records to consolidate the extracted information and discuss discrepancies in the charted information.

### Collating, summarising, and reporting the evidence

This stage was implemented following the three steps outlined by Levac et al. (2010), which includes analysis, reporting the outcome of analysis, and relating the results to the review’s purpose and its potential practical implications. Analysis of charted data was organised in two ways. First, numerical representation of the extent, nature, and distribution of application of sociomaterial perspectives in IPE and IPC was summarised. Findings were mapped to provide an overview of health professionals involved in the study, geographic distribution of where the studies had been implemented, and the range of sociomaterial applications described. Organising data in this manner provided not only an indication of the most common applications of sociomaterial theory in IPE and IPC, but also the gaps in knowledge on the topic. Second, we thematically organised the studies. Themes were developed using an inductive iterative approach. Two reviewers performed initial categorisation, which was verified and refined together with two other researchers in the team. The findings of this scoping review were interpreted based on the application of the sociomaterial theory in IPE and IPC.

## Results

**Fig. 2:**
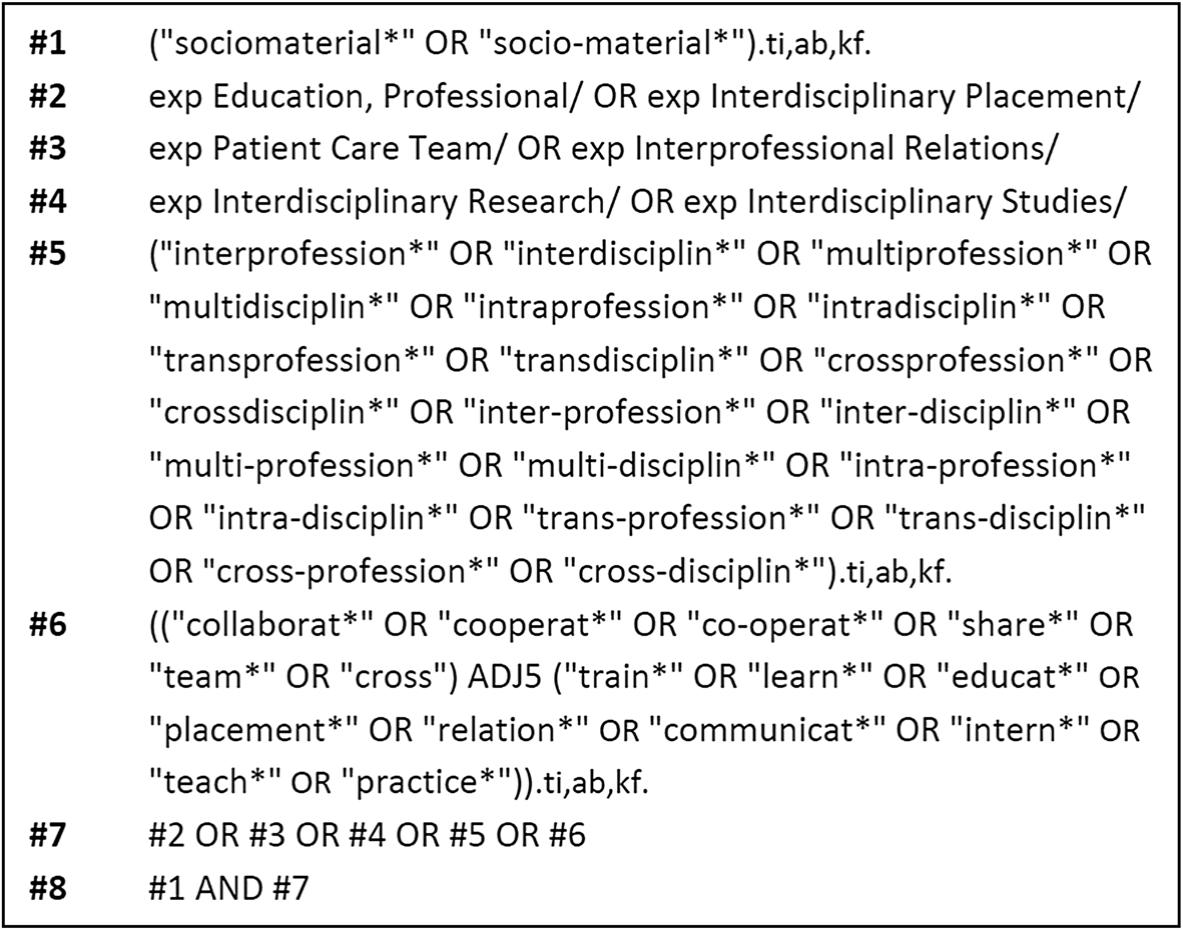
Sample search strategy for PubMed

After deduplication, the database search in September 2021 yielded 250 records. Following title and abstract screening, 58 records remained, of which 23 were excluded after full-text screening. From the citation search of the 35 included articles, we further identified 8 records, bringing up the total number of included papers to 43 (Fig. 2). Included records are referenced using the superscript provided and are numbered alphabetically (Table 1).

**Table 1:** Summary of records (*N* = 43) included in the scoping review

#	References	Type of PUBLICATION	Study design	Country	Setting	Included professions	Specified theories under socio-materiality
1	Ahn et al. (2015)	Original research	Qualitative (ethnography)	Sweden	IPE at a clinical skills training and simulation centre	Students: medicine, nursing	Actor-network theory
2	Ahn and Rimpiläinen (2018)	Original research	Qualitative (observational study)	Sweden	IPE in a simulation training	Students: nursing, medicine	Actor-network theory
3	Beane & Orlikowski (2014)	Conference proceeding	Qualitative	USA	IPC in a post-surgical ICU at a teaching hospital	Professionals: medicine, nursing	None
4	Bleakley (2010)	Discussion paper	NA	UK*	IPE in medical education	Professionals: medicine, nursing	Complexity theory, Communities of practice/situated learning, Actor-network theory, Cultural-historical activity theory
5	Bridges et al. (2020)	Original research	Qualitative (ethnography)	Hong Kong/ Macau	IPE in a interprofessional team-based learning programme	Students: medicine, nursing, pharmacy, medical laboratory science, OT, PT, radiography, and social work	none
6	Burm et al. (2019)	Original research	Qualitative (grounded theory)	Canada	IPC in an inpatient internal medicine teaching unit	Mixed professionals and students: medicine, nursing	Actor-network theory
7	Byerly et al. (2021)	Original research	Qualitative (longitudinal case study)	USA	IPE in an elective placement (based at a nursing home)	Students: medicine, pharmacy, PT	Activity theory
8	Dahlgren et al. (2012)	Book chapter (chapter 12)	NA	Sweden	IPE in professional healthcare education	Students: medicine, nursing, biomedical laboratory science, OT and PT	Practice theory
9	Dalinghaus et al. (2021)	Original research	Qualitative (exploratory case study)	Canada	IPE during a videoconference-enabled simulation and debriefing	Professionals: medicine, nursing	Critical theory
10	Dupret and Friborg (2018)	Discussion paper (with case examples)	NA	Denmark	IPC in technology workarounds as initiatives of tacit innovation	Professionals: medicine, nursing, information-technology	Actor-network theory, Social-constructionist language theory
11	Husebø et al. (2019)	Book chapter (chapter 7)	NA	Sweden, Norway, Denmark*	IPE during debriefing in simulation activities	Professionals and students: medicine, nursing	Practice theory, Experiential learning theory
12	Essen et al. (2015)	Discussion paper	NA	UK*	IPC within the hegemony of psychiatry in the field of mental health	Professionals: medicine, nursing	Distributed cognition theory; Personal construct theory
13	Falk et al. (2017)	Original research	Qualitative (ethnography)	Sweden	IPC at a spinal cord injury rehabilitation unit	Professionals: medicine, nursing, OT, PT, rehabilitation assistant	Practice theory, Cultural-historical activity theory, Expansive learning
14	Falk et al. (2017)	Original research	Qualitative (ethnography)	Sweden	IPC at a spinal cord injury rehabilitation unit	Professionals: medicine, nursing, OT, PT, rehabilitation assistant	None
15	Fenwick (2012)	Discussion paper	NA	UK*	IPE and IPC in diverse contexts like management, social service, health care and education	Professionals and students from a range of professions/disciplines (e.g., public health, management science, environmental science, medicine, nursing)	Complexity theory
16	Fenwick (2014a, 2014b)	Original research	Qualitative (case study)	UK	IPC in emergency mental health care	Professionals: paramedicine, law enforcement, nursing, medicine (accident & emergency consultant)	Actor-network theory, complexity theory, spatial theory, new materialist, posthuman analyses
17	Fenwick (2012)	Book chapter (chapter 7)	NA	UK*	IPC/Co-production in diverse context	Professionals: variety of health and non-health professions/disciplines	Complexity theory, Communities of practice, Model of relational agency
18	Goldszmidt and Faden (2016)	Commentary	NA	Canada*	IPE in medical education	Non-specific	Actor-network theory, cultural-historical activity theory, Communities of practice
19	Hibbert et al. (2018)	Original research	Qualitative (Critical narrative inquiry)	Canada	IPC at a clinical teaching unit (based on experiences around an elderly patient)	Professionals: medicine, nursing, social work	Actor-network theory
20	Hopwood et al. (2019)	Book chapter (Chapter 5)	NA	Australia*	IPE in using simulation as an educational tool	Students: nursing and medicine	Actor-network theory
21	Jorm et al. (2016)	Original research	Mixed-methods	Australia	Developing a student-directed IPE activity	Students: diagnostic radiology, exercise physiology, medicine, nursing, OT, pharmacy, PT and speech pathology	Complexity theory
22	Koivisto et al. (2015)	Discussion paper	NA	Finland	IPC at different levels of actors in an innovation project	Professionals: Social work, medicine, nursing, information technology	Relational ontology, Actor-network theory, Social theory
23	Kvarnström et al. (2018)	Original research	Qualitative (ethnography)	Sweden	IPC in several surgical units	Professionals and students: nursing, medicine	None
24	McMurtry (2013)	Discussion paper	NA	Canada*	IPE / IPC	Non-specific	Communities of practice, Cultural-historical activity theory, Complexity theory, Actor-network theory
25	McMurtry et al. (2016)	Review paper	NA	Canada*	IPE	Non-specific	Communities of practice, Cultural-historical activity theory, Actor-network theory
26	Melo and Bishop (2020)	Original research	Qualitative (case study)	Portugal	IPC for a fall prevention programme in a large acute teaching hospital	Professionals: medicine, nursing, engineering, social work, and laboratory science	Normalisation process theory, Theory on material infrastructure, Actor-network theory
27	Möller et al. (2022)	Original research	Qualitative (case study)	Sweden	IPE in a clinical placement (based at an orthopaedic ward)	Students: medicine, nursing, PT, and OT	none
28	Nyström et al. (2016a)	Original research	Qualitative (observational)	Sweden	IPE during debriefing sessions following simulation activities	Students: medicine, nursing	Practice theory
29	Nyström et al. (2016b)	Original research	Qualitative (ethnography)	Sweden	IPE during simulation activities	Students: medicine, nursing	Practice theory
30	Nyström et al. (2016c)	Original research	Qualitative (exploratory case study)	Sweden	IPE during the observation of simulation activities	Students: medicine, nursing	Practice theory
31	Nyström et al. (2017)	Original research	Qualitative (case study)	Sweden	IPE during a simulation-based training for teamwork and IPC (as part of continuing professional development)	Professionals: medicine, nursing, midwifery	Practice theory
32	Oates (2016)	Commentary	NA	Australia*	IPE in the pre-qualification education of health professionals	Non-specific	Communication theory, Team role theory
33	O’Brien et al. (2017)	Commentary	NA	USA	IPE during simulation activities	Non-specific	none
34	O’Leary et al. (2021)	Discussion paper	NA	Ireland*	IPE in placements for students of allied healthcare programmes	Non-specific	Complexity theory, Actor-network theory, Structuration theory, Activity theory
35	Pedersen (2012)	Book chapter (chapter 2)	Qualitative (interpretive)	Norway	IPC during computer-mediated nurse-nurse handover in a cardiology ward	Professionals: medicine, nursing	None
36	Schindel et al. (2019)	Original research	Qualitative (comparative case study)	Canada	IPC in community pharmacy practice	Professionals: pharmacy, medicine, nursing, and other healthcare professions	Document theory
37	Sergeeva et al. (2015)	Conference proceeding	Qualitative (ethnography)	Netherlands	IPC during robotic surgery at a surgical unit	Professionals: medicine, nursing	Practice theory
38	Stewart (2014)	Original research	Qualitative (ethnography)	Australia	IPC / interagency work in domestic violence	Professionals: legal, social and health professions	Actor-network theory, Cultural-historical activity theory, Expansive learning
39	Stewart (2020)	Original research	Qualitative (ethnography)	Australia	IPC / interagency work in domestic violence	Professionals: legal, social and health professions	Actor-network theory
40	Thompson et al. (2018)	Original research	Qualitative	Canada	IPE in undergraduate medical education	Students: medicine	Complexity theory
41	Thörne et al. (2017)	Original research	Qualitative	Sweden	IPC during ward rounds at an inpatient internal medicine unit	Professionals: medicine, nursing	Practice theory
42	Vuojärvi and Korva (2020)	Original research	Qualitative (ethnography)	Finland	IPE during simulation training at a hospital	Professionals: medicine, nursing	Leadership-as-practice theory, Cultural-historical activity theory
43	Waelli et al. (2016)	Original research	Qualitative (ethnography)	France	IPC during implementation of a national quality indicator in healthcare organisations	Professionals: medicine, nursing, information technology	Cultural-historical activity theory

*IPE* interprofessional education, *IPC* interprofessional collaboration, *OT* occupational therapy, *PT* physiotherapy

### Mapping out the included citations

Majority of the included papers were original research articles (*n* = 25) while the rest of the papers were discussion papers (*n* = 7), book chapters (*n* = 5), commentaries (*n* = 3), conference papers (*n* = 2), and a review (*n* = 1). Twenty-eight papers (research articles = 25; conference papers = 2; review = 1) presenting empirical data employed qualitative methods, with one paper using a mixed-methods design. Many of the papers (26 out of 43 papers) were from Europe, with Sweden (*n* = 13) and the United Kingdom (UK) (*n* = 5) as the second and third rank, respectively. Papers from the Americas were from Canada (*n* = 8) and the United States (US) (*n* = 3). There were also papers from Australia (*n* = 5) and Hong Kong/Macau (*n* = 1).

Out of the 43 articles, seven were not aligned with Orlikowski’s definition due to the following reasons: unconventional terminologies describing sociomaterial concepts^3,7,10,12^, the included literature was a book chapter which may have captured the definition elsewhere within the book^17^, and a paper where no explicit definition was provided^43^.

We observed variations in authors’ definitions of IPE and IPC and identified distinctions in the terminologies used. Only 22 of the 43 articles aligned with either the WHO (2010) or JIC (n.d.) definitions of IPE and IPC. Of the 21 that did not align, five of the included articles defined and focused on IPC, and so did not elaborate on IPE^4,6,12,13,14^. Other records were either unclear in their definitions of (but only alluded to) IPE and/or IPC^1,2,3,11,16,20,38^, or gave examples rather than definitions^25^. Additionally, some records used distinct terminologies pertaining to IPE, such as “workplace learning” ^19^ and “collaborative learning”^22^. Other terminologies alluding to IPC included “interprofessional practice”^16^, “interprofessional co-creation”^22^, “interdisciplinary endeavour”^22^, “interdisciplinary collaboration”^35^, and “interagency collaboration”^39^. The remaining records defined neither IPE nor IPC^9,10,26,27,36,37,42,43^.

**Fig. 3:**
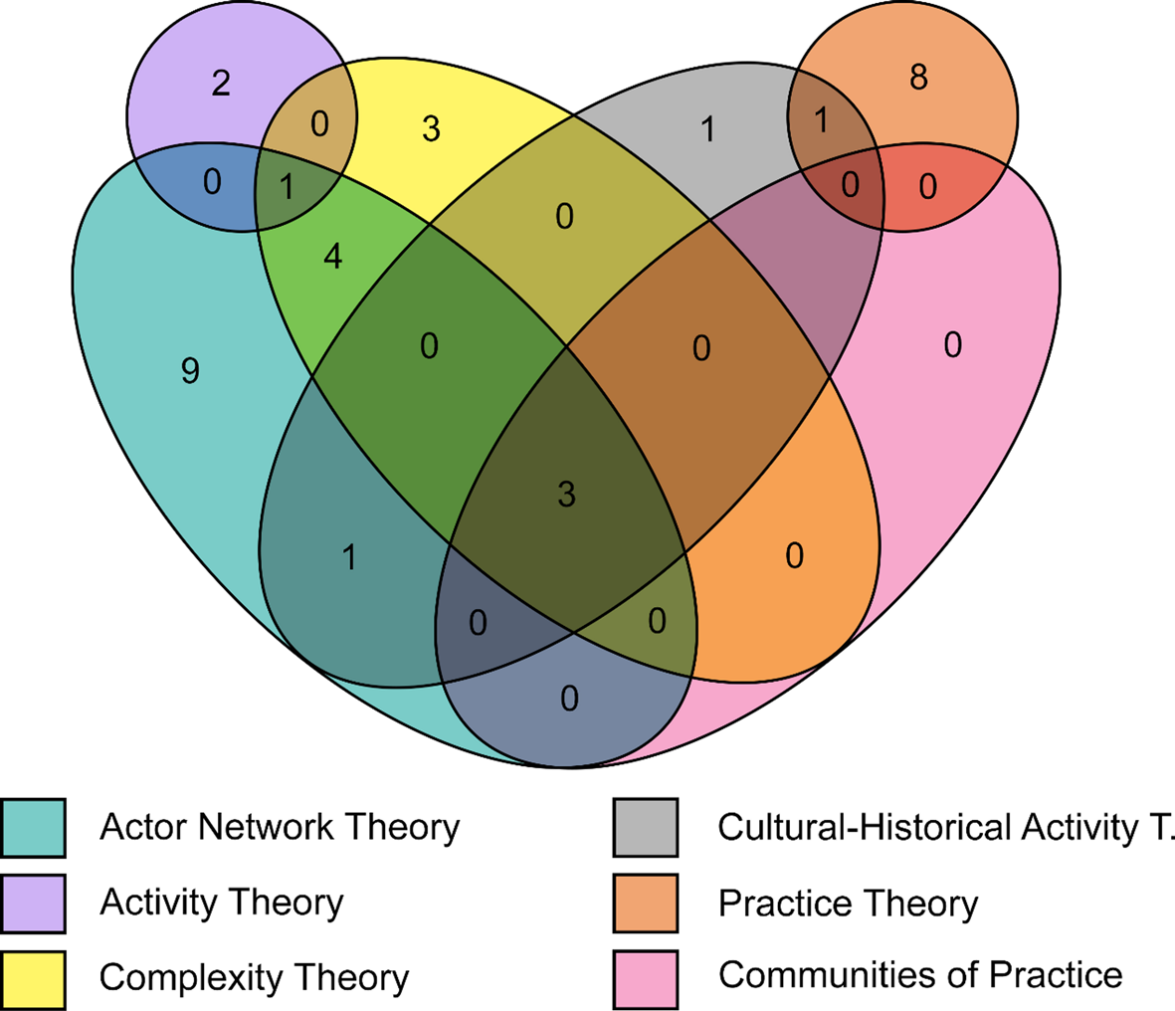
Sociomaterial theories Venn diagram

Thirty-three records utilised theories under the sociomaterial perspective. ANT appeared most frequently among our included literature^1,2,4,6,10,16,19,20,22,24,25,26,34,38,39^. Other related theories (under this perspective) included complexity theory^4,16,17,21,24,25,34,40^, CHAT^4,14,24,25,38,43^, activity theory^7,34,42^, practice theory^8,11,14,28,29,30,31,37,41^, and CoP theory^4,24,25^. Among the 33, only three records have mentioned and/or utilised the four main sociomaterial theories^4,24.25^ (Fig. 3). Moreover, there were records that referenced and utilised theories that shared common elements with sociomateriality. These included relational ontology ^22^, normalisation process theory ^26^, theory on ‘material infrastructure’ ^26^, team role theory ^32^, presage-process–product (3P) of the student learning theory^34^, structuration theory^34^, document theory^36^, leadership-as-practice theory^42^, critical theory^9^, and Gergen’s social-constructionist language theory^10^. Other theories from various disciplines used to frame a sociomaterial lens included critical realism^12^, distributed cognition^12^, and personal construct psychology^12^. Theories of learning were also referenced and applied using sociomaterial concepts, especially within those included records that focused exclusively on IPE. These included Engeström’s expansive learning theory^14,38^ and Kolb’s experiential learning theory^11^. Specific proponents closely associated with their advocated theories were also labelled in some of the included literature, which included Schatzki’s practice theory^8,14,31,42^, Gergen’s social constructionism^10^, Mol’s iteration of Latour’s ANT^38^, and Mol’s work on multiple ontologies^20^.

Nineteen articles primarily discussed the application of sociomaterial practices in IPE and IPC^1,2,11,17,20,23,25–34,36,37,41,42^. These studies explained health and social care practices from a sociomaterial perspective: the use of mannequins, medical equipment, technology, and physical spaces as crucial amplifiers in establishing the bi-directional relationship between the social and the material. Eighteen studies used sociomateriality as a theoretical lens to ground the study design^5–9,12,13,15,16,19,21,23,24,34,35,38,40,43^, eleven of which used sociomateriality as a framework for the analysis of their data^5–8,12,15,24,35,39,40,43^. Other studies contributed salient insights on specifying concepts, phenomena, and realities of sociomateriality^10,13,18,22,24,25^ such as knowledge sharing and co-creating processes of collaborative learning^18,22^.

Twenty-one studies presented the development of group formation and social processes through configuration of learning setups and human and non-human regulatory processes^3–7,10,16,17–19,22,24–27,33,36–39,42^, three of which focused on sharing of practices with other organisations and/or agencies^16,38,39^. Two studies illustrated the roles of nurse practitioners and clinical managers^23,43^, while other studies presented findings related to sociomaterial learning activities intertwined with practical clinical application^13,14,29,30,32^, reflection of IPC during learning activities employing sociomaterial approaches^28,31^, and methods in facilitating interprofessional learning employing concepts of sociomateriality^1,21,41^.

### Thematic analysis

Themes derived from the scoping review included (1) power as a sociomaterial entity shaping IPE and IPC, (2) inclusion of non-health professionals in reimagining IPE and IPC practices, and (3) the misconception of sociomateriality.

*Theme 1: Power as a sociomaterial entity shaping IPE and IPC.* Several power-related conceptualisations were identified in this scoping review. One example is the discourse on power sharing or “depowering,” where health and social care professionals involved in IPE and IPC are expected to breach their usual professional boundaries to accommodate multiple levels of collegiate sharing (D’Amour & Oandasan, 2005). According to Essen et al. (2015), power is socially constructed, and social power has its material basis: “Power is entrenched in layers of material investment habituated in human behaviour and resistive artefacts that afford future actions and limit others” (Essen et al., 2015, p. 215). For instance, wearing uniforms (materiality) influences power within health care teams. In some contexts, wearing longer, white coats denote having a higher position in the hospital among doctors, while those who do not wear white are perceived to have lower positions. Recently, most health care professionals have started wearing the same scrub suits, where the only distinction would be the name plate, to decentralize the power across the health care team.

We also need to recognise that the locus of power shifts from one actor to another within the networks where materials and humans interact as demonstrated in interagency domestic violence work (Stewart, 2014). Where power relation discourses entail talking about conflicts and hierarchies, the medical profession continues to reject the relevance of social learning theories that posit how knowledge is shared and commonly owned. In the clinical education and practice, these contemporary theories underscore the idea that knowledge must be shared in order to identify problems and generate solutions as a team through collaboration, democratization, and horizontalization of power (Bleakley, 2010). These principles were argued and rejected broadly by the medical profession because of its “tradition of heroic individualism where knowledge is treated as a private capital” (Bleakley, 2010, p. 849). Consequently, a heightened awareness of power relationships can enhance the operationalization of IPE and IPC practices (Dalinghaus et al., 2021). If uncritically examined, power can be used to deceive via what Freshwater et al., (2014, p. 65) call as “role violation” and “dysfunctional consonance”. These conceptualisations characterise scenarios where interprofessional practice gives the outside impression of “collaborative success” whilst tacitly empowering one group to continue its paradigmatic dominance over others (Essen et al., 2015, p. 218).

*Theme 2: Inclusion of non-health professionals in reimagining IPE and IPC practices* The second theme that emerged was on how non-health professionals contribute to the primary goal of IPE and IPC: to engage in collaborative learning and working towards achieving improved health outcomes for individuals, communities, and populations. These non-health professionals included professionals in systems dynamics, knowledge management, tobacco control, management sciences and health policy (Fenwick, 2012); police (Fenwick, 2014a, 2014b; Stewart, 2020); engineers (Melo & Bishop, 2020); information technology developers (Koivisto et al., 2015); head of information systems department (Waelli et al., 2016); IT professionals (Dupret & Friborg, 2018); operational managers (Stewart, 2014); quality managers (Waelli et al., 2016); and porters (Vuojärvi & Korva, 2020). These non-health professionals tend to be viewed as paraprofessionals involved in work arrangements resulting in cross-practice relations, opening boundaries, and expansion of clinical judgement or decision-making in the health care setting (Fenwick, 2014a, 2014b). The expanded sociality gained between health and non-health professionals allow for more complex materials that are needed to address or achieve wicked problems in health sciences education and health care practice.

*Theme 3: The critical understanding of sociomateriality* This last theme revealed a subjective and socio-historico-political understanding that aims to facilitate a dialectical discourse on sociomateriality. Our results show that 42 out of 43 papers came from Westernised countries, with only 1 paper coming from Hong Kong/Macau, a past colony of the UK for more than 150 years. The results of the published article from Hong Kong/Macau present the effect of interwoven assemblages of the social and material learning environments on developing cohesion of interprofessional teams (Bridges et al., 2020). Whilst the term ‘Westernised’ varies based on context, geographically, Western nations constitute the majority of Europe, North America, and Oceania. With this, we would likely assume that experts and authors of sociomateriality in the context of IPE and IPC are broadly influenced by Western philosophies and ideologies that focus on individuality, universality, and formality. However, this presumption is contradictory to what we found in this review since the sociomaterial perspective had been used as a perspective to reject the conceptualization that man is central, and the truth is formal and universal. These scholars examined IPE and IPC contexts using a sociomaterial perspective in order to critique their own knowledge and seek to evolve and develop new ones.

## Discussion

This scoping review’s purpose was to map the breadth of literature on the application of sociomaterial perspectives to IPE and IPC. We identified 43 articles that originated mostly from Western countries and included original research articles, discussion papers, book chapters, and other types of documents. Salient findings will be discussed: reimagining IPE and IPC through sociomateriality, discussing power as a sociomaterial entity that shapes IPE and IPC, and applying sociomateriality in IPE and IPC practices.

### Reimagining IPE and IPC through sociomateriality

To reimagine is to rethink concepts and practices from a different lens. Sociomateriality, initially introduced as a free-standing theory, has evolved into a much broader concept encompassing a range of other prominent postulates and perspectives. Based on our findings, we describe here selected theories (written in bold text) based on their sociomaterial characterisation and how their tenets are and can be utilised in IPE and IPC practices.

CoP and ANT (Bleakley, 2010; Latour, 2005; McMurtry, 2013; McMurtry et al., 2016) position learning as being a product of agential interactions and exceeding that of isolated metacognition, with CoP being focused more on the sociality aspect. Despite their differences, both theories consider a social organisation as constitutive of health professionals and/or students who share a similar goal of improving the quality of patient care. Grounded on an anti-reductionist commitment, these theories consider the importance of both social and material entities. To exemplify, the theory of material infrastructure (Nicolini et al., 2012; Melo & Bishop, 2020) regards materials as an iterative and “naturally occurring” assemblages in collaboration. This indicates that even objects as specific as documents (e.g., medical records) may be ushered towards saliency within a sociomaterial perspective, as the document theory (Lund & Skare, 2010; Schindel et al., 2019) posits that documents have three properties that are both intrinsic and inseparable: the material, the social, and the mental. In addition, relational ontology (Latour, 2005; Koivisto et al., 2015) propounds the fundamental ontology of the social and material. Thus, the working relationship between two health professionals and how they navigate within materialities in a specific setting further enhances the primacy of both entities, as compared to when the material and social are working in a parallel but disjointed space. A sociomaterial perspective grants us the humility to accept that without materialities, we cannot achieve our professional and human goals. The sociomaterial arrangements of educational activities, such as virtual and physical learning environments, prefigure a practice of IPC between future health professionals (Dahlgren et al., 2012). Identifying spatial and social settings of learning environments (i.e., hospital wards, simulation rooms) influences co-productive learning activities (i.e., hospital ward rounds), integrates quality improvements in the health professionals’ knowledge and delivery of care (Thörne et al., 2017), and reconceptualises professionals’ learning in response to the changing practice landscapes (Oates, 2016).

Complexity science (Cilliers, 1998; McMurtry, 2013) also embraces the notion of ‘social organization’, but uses the term ‘systems’ instead, to describe a group of health care professionals learning with each other by. For example, interacting in a busy operating theatre, or in an interprofessional classroom attended by students from different fields, in a complex setting bounded in purposeful ‘chaos’. Within the complexity science viewpoint, the system cannot be merely explained by deterministic approaches that only consider the human’s individualised aspect of learning and collaboration. In other words, learning must be viewed as a complex and adaptive system consisting of numerous elements, also known as “more than the sum of the parts”. For instance, Newell (2008) described the ‘class’ as a self-organising entity that is within a complex and adaptive system, which must be the locus of learning and teaching rather than the individual student. Grounded on complex adaptive systems, IPE classes can benefit from using small group and problem-based learning arrangements (Mennin, 2007), thereby the competence of working in a health care team must be taught, trained, and measured as a collective rather than as an individual competence (Lingard, 2012).

CHAT (Engeström, 2001; McMurtry, 2013) offers an expansive glimpse into activity systems that also play a role in everyday health care practice. Within the activity system are activity factors: the subjects, objects, mediating artefacts, social rules, and even the layout and structure of the physical environment where the learning and collaboration takes place. Similar to complexity science, a ‘system’ is composed of activities that are interconnected. The interplay of factors within an activity system is crucial to historically situate and produce intended or unintended outcomes such as learning together, providing care as a team, or conflict resolution. Similar to CHAT, the ‘presage’ aspect of the 3P theory (Biggs, 1993; O’Leary et al., 2021) considers not just the student but also the teaching context, i.e., the classroom setting, pedagogy, and use of educational technologies. Doing so allows for meta-learning or the process of ‘learning to learn’ to occur in order to proceed with the ‘process’ and ‘product’ aspects of the 3P theory. In this case, meta-learning could happen when students and professionals are taught how to learn the process of learning with others within the context of improving health care delivery and outcomes. To do this concretely, health professions educators and curriculum developers must facilitate clinical decision-making through group learning opportunities (Berger et al., 2022).

Some identified theories highlight the interplay between the social and material entities. For instance, normalisation process theory (May & Finch, 2009; Melo & Bishop, 2020) intends to elucidate how and why new health practices can be embedded (‘normalised’) within everyday work in a social organisation. While IPE and IPC are not new concepts, introducing them (alongside new techniques and technologies) within health systems will require coherence (*What are the purposes of IPE and IPC?*), cognitive participation (*What promotes the participation in IPE and IPC initiatives?*)*,* collective action (*How do participants work together to make IPE and IPC work within the organization?*), and reflexive monitoring (*How do the participants appraise these IPE and IPC initiatives?*).

Speaking of participants working together, the team role theory (Belbin, 2012; Oates, 2016) posits that successful and high-performing teams are composed of people that, despite behaving in different ways, can work collectively. These teams, however, are governed by social structures that influenced the team’s actions. The structuration theory (Giddens, 1984; O’Leary et al., 2021) explains this further by specifying the importance of where actors (e.g., health profession practitioners and students) operate within contextual rules and social structures. For instance, interprofessional teams working in different settings may have a shared goal of providing safe and quality health access to patients but achieving it may require different actions dictated by the rules, cultures, and bureaucratic processes within their respective organisations. One team might be required to work with electronic medical records (EMR) to coordinate communication, whereas another team may only use EMR as an option because their organisation prefers regular on-site meetings.

The notion that the world and events are the same for all, but humans and groups respond and observe them differently, is an assumption that is clearly explained by the personal construct psychology (PCP) (Essen et al., 2015; Kelly, 1955; Winter, 2017) and distributed cognition theory (Essen et al., 2015; Hutchins, 1995). Similar to the conceptualisations of IPE and IPC, team members from different professions are intentionally grouped in the same situation with a shared goal. However, we cannot discount the fact that their responses to the situation, in this case patient care, may differ from each other. This may be due to their personal constructs (as in PCP) or cognitive psychology (as in distributed cognition theory). For instance, during patient evaluation, a physician might require a catheter, the nurse would look into monitoring the patient's catheter use every hour, while an occupational therapist would seek ways to enhance the patient’s skill in transferring from bed to the bathroom and vice versa, in consideration of the catheter. Regardless of the differences, there is one patient that they need to attend to, underpinned within the shared goal of patient safety and quality care. Language use and dialogue also play a significant role. From a social-constructivist viewpoint and based on the social-constructionist language theory (Dupret & Friborg, 2018; Gergen, 1985), dialogue about the world is seen not merely as a mirror image of itself but as a product of continuous communicative exchanges enmeshed and sustained by surrounding materialities.

Akin to the theories mentioned prior, CoP (Lave & Wenger, 1991; McMurtry, 2013) espouses three familiar elements: domain (shared interest on IPE and IPC), community (members who pursue this shared interest), and practice (products of the domain made by the community including repertoire of resources, ideas, or stories). While having a *shared interest* is crucial in a community of practice, we cannot discount the fact that not all will have the same interest in IPE and IPC. Hence, going beyond networking with other members of the team is warranted, and this can lead to ‘knotworking’. Knotworking activities go beyond socializing and interacting among practitioners as these entail them to view collaboration as constantly changing and requiring continuous negotiation or ‘shaking of structures’ according to the shifting needs, interests, and concerns in health care delivery. Ideally, these negotiations produce a *practice* reflected on actions, projects, and policies.

Among the theories described, there is a pivot in foci: from solely looking into the social discourses between people and groups to foregrounding the contribution of material assemblages (settings, bodies, and devices) to actuate IPE and IPC practice arrangements (Fenwick, 2014a). These practices include creating laws and policies, enabling and navigating practice guidelines, and developing and achieving learning outcomes (Ahn & Rimpiläinen, 2018). Part of this reimagination is the conscious use of orthography where the terms ‘sociomateriality’ and ‘interprofessional’ are spelled without hyphenation. Doing so affects the lexical semantics (meaning of words) of how these terms are used and practised, i.e., for sociomateriality, the social and material are perceived to be inseparable, and for interprofessional, professionals indeed interact.

### Power as a sociomaterial entity that shape IPE and IPC

The identified theories in this scoping review—one way or another, explicitly and tacitly—highlight the material basis of social power to explain the emergence of knowledge, the orchestration of practice processes, and the transaction of information between people and materialities within the ecology of professionalisation (Essen et al., 2015; Falk et al., 2017). Within the interprofessional field, the issues and concepts of power have recently been discussed (Konrad et al., 2019). Power in healthcare practice is entrenched within layers of material investment grounded in human behaviour (Essen et al., 2015) and the degree of participation of actors within practice architectures (Pedersen, 2012). Power is then seen both as an enabler and barrier in achieving activities and fostering relationships between professionals (Falk et al., 2017). Therefore, it is crucial that efforts towards IPE and IPC practices are investigated under the sociomaterial lens where social and material entanglements are foregrounded over uniprofessional and standardised practice arrangements (i.e., nurse-nurse handover). The dynamics of sociomaterial constructions illuminate the complexities of collaborative practice (Kvarnström et al., 2018). These include power dynamics and conflict resolution among team members (Fenwick, 2012), deception of power wherein IPE and IPC practices are being subtly used to reinforce the dominance of the medical profession (Freshwater et. al., 2014), challenging the boundaries of disciplinary and professional knowledge to form new ways of knowing (Hopwood et al., 2019) and identifying repressive conditions within professional practice (Fenwick, 2012).

Speaking of power, this scoping review also revealed that the included papers largely come from higher-income, Western nations with global influence. However, our analyses ascertained that we cannot presume that their conceptualisations were largely influenced by Western philosophies. In fact, their arguments sought to challenge the dominant assumptions on individualism and finding the ‘truth’. Themes on sharing a common goal, enabling collective actions, viewing entities as a whole rather than separately, and considering pluralistic viewpoints among others have emerged which are not necessarily Westernised. Although all authors of this paper are non-Western, we hope to support the argument that from our perspective at least, sociomaterial perspectives cannot be confined in Western theorisations alone.

### Applying sociomateriality in IPE and IPC practices

Sociomateriality has five key notions as outlined by Orlikowski and Scott (2008) (Jones, 2014): materiality, inseparability, relationality, performativity, and practice. *Materiality* does not only talk about ‘stuff’ that are solid and tangible, but also those that are intangible including data and algorithms. *Inseparability* connotes the coalescence of the social and material ascertaining that “Materiality is thus viewed as integral to human activity and there is no social action that does not entail material means” (Jones, 2014, p. 898). In spaces where IPE and IPC are enacted, tangible materials are interpenetrated within human activities such as using sticky notes to generate a collaborative care plan in a case-based study group and sharing of medical charts among nurses and occupational therapists in an acute psychiatric ward. *Relationality* means that humans and technologies only exist in relation to one another. For instance, IPE and IPC cannot occur when there is no interaction between health professionals (human entities) and teaching–learning tools and medical instruments. Although there are many ontological differences in explaining the relationality between the social and material, we need to recognize that while inseparable, they are not symmetrical in terms of properties. *Performativity* denotes that objects are stable and neutral material entities such as “institutions, financial instruments, technologies, decision making, design, service delivery, strategies and discourses” (Horan et al., 2014). While innately stable, they can become unstable, immutable, and agential when these objects are intertwined with human entities resulting in ‘performances’ including social outcomes, transformations, and even resistance. For example, health service delivery protocols are materialities that can either improve or diminish the job performance of healthcare workers depending on how these are applied or adhered to. *Practice*, in the context of sociomateriality, is defined as the formation of activities constituting the use of ‘things’ and the aggregates of doings, knowings, feelings, and sayings (Reckwitz, 2002). The ultimate goal of IPE is to produce a collaborative-practice ready health workforce who will engage in ‘collaborative practice’ (IPC). While social phenomena and overt behaviours have been the standard in measuring ‘successful practice’ in IPE and IPC, foregrounding the spatial and affective aspects of these social phenomena is warranted through means of qualitative and mixed method research underpinned by sociomaterial perspectives.

Through the sociomaterial lens, we can see how IPE, IPC, and health professions education are co-constructed by different theories, and their derivatives, beyond the fields of education and healthcare. The WHO has mapped the classification of health workers based on the International Labour Organisation (2008), which include all types of health-related occupations as well as the occupation group ‘non-health professionals not elsewhere classified’. They are described as professionals that work within the health systems such as (but not limited to) physical, mathematical and engineering science professionals, teaching professionals, business and administration professionals, information and communications technology professionals, legal professionals and social science professionals. Malcolm Cox during the global forum on the Innovation in Health Professional Education (Washington DC in 2015) included in his summarization of lessons learned that “it is wrong to think of community health workers as non-professionals” (National Academies of Sciences, Engineering, and Medicine, 2016). He then proposed that the people (frontliners and community health workers) who form the base of the health pyramid should be considered professionals contributing to the operation of the health system (ibid.). Anchored on this realization, our findings ascertain non-health care practitioners contribute, directly or indirectly, in actuating IPE and IPC practices.

Lastly, we found that a sociomaterial perspective is mainly used as a theoretical framework to situate and deconstruct IPE and IPC practices. Since this study aimed to foreground the equivalency of materiality to the social aspects of interprofessional education and collaborative practice, we espouse the consideration of sociomaterial perspective aside from the more common perspective drawn from social constructionism (artifacts/materials are produced from group/social interaction) and social constructivism (individual learning occurs as a result of social interaction). It is also utilised to guide research designs and data analyses, specifically those that use mixed method and qualitative methodologies. For example, using a sociomaterial lens can enhance the interpretation of cohort studies using standardised tools such as the Collaborative Practice Assessment Tool-Revised (Sy et al., 2019). While we recognise that conflict and resistance drawn from the existence of professional hierarchies and silos are inevitable, utilising a sociomaterial lens can help IPE and IPC scholars and practitioners to deconstruct the ‘why’ and the true meaning of interprofessionalism within specific health care contexts.

The results of this scoping review are limited in its capacity to make strong conclusions regarding IPE and IPC as framed in a sociomaterial perspective. Rather, our results support the need to cultivate an on-going discourse on the topic. The added value of this paper is that we were able to map out current publications on the topic under study as a review, which has not been done before. Although one may argue that a more granular search of the different aspect of sociomateriality may have given more information, that kind of review would have required more resources (e.g., time, money, people) to implement. Provided that this research was self-funded with only five people in the research team, we recommend that in the future, a more systematic and robust review can be done, considering this scoping review as a guide. Nevertheless, our results can aid policymakers in determining as to whether a full systematic review is warranted or not, as well as help practitioners in devising practice guidelines in healthcare and health professions education utilising perspectives beyond statutory, dominant, and reductionist approaches.

## Conclusion

This scoping review provided a clearer understanding of how a sociomaterial perspective is being applied within IPE and IPC practices. There is no question that sociomateriality has been used to frame numerous studies in different ways in the past 15 years. While theories associated with sociomateriality have been highlighted (i.e., ANT, complexity theory, CHAT, practice theory, and CoP), more theories with sociomaterial characteristics were unearthed. The sociomaterial perspective was defined and described based on Orlikowski’s (2007) original works. However, efforts to have a unified definition of the concept were not apparent, which was supported in the editorial written by Thistlethwaite and Xyrichis (2022, p. 165): “There is a risk that IPE focuses on a narrow definition of teams and teamwork that does not enable students to observe, discuss and participate in the complexity of practice as enacted in their context.” Although one thing is for sure, the use of a sociomaterial perspective within IPE and IPC contexts underscore the existence of material entities as equal players to social entities that contribute to the formation of practices.

The growing complexity of practices within healthcare contexts necessitate the examination of not only overt social and material entities but also covert links that entangle them. To do that, sociomateriality is a viable perspective that can be used, applied, and even exploited to understand complex issues in healthcare such as power relations, hierarchies, pandemics, climate change, shortage of human resources for health, emergency remote teaching, and patient safety among others. The intentional use of sociomateriality as a perspective espouses a deeper and broader understanding on how IPE and IPC practices are developed or dissolved. For instance, while not aligned to the traditional definitions of IPE and IPC, our results lead to recommending the inclusion of non-health professionals and material arrangements to shape present and future IPE and IPC practices. Acknowledging this potential can galvanise the possibility of actuating as well as expanding new and emerging roles in health care (Fraher & Brandt, 2019). Moreover, power relations in IPE and IPC practices can be seen as a sociomaterial entity that is often ignored without putting on a sociomaterial lens. This scoping review underscores the need to emphasise material entities as factors that influence learning and working practices especially in contexts where multiple professionals, cultures, and settings are involved. Lastly, recognising that human and non-human entities—including its known and unknown links, boundaries, and nexuses—constitute the complexity and ‘messiness’ of IPE and IPC practices which can prepare us for the new professionalism and collaborative entanglements that await us in the future.

### Supplementary Information

Below is the link to the electronic supplementary material.

Supplementary file1 (DOCX 16 kb)
